# Treatment delay among pulmonary tuberculosis patients in pastoralist communities in Bale Zone, Southeast Ethiopia

**DOI:** 10.1186/1756-0500-5-320

**Published:** 2012-06-21

**Authors:** Awol Hussen, Sibhatu Biadgilign, Fasil Tessema, Shikur Mohammed, Kebede Deribe, Amare Deribew

**Affiliations:** 1 Bale Zonal Health Department, Oromia Region, Ethiopia; 2 Departments of Epidemiology and Biostatistics, Jimma University, Jimma, Ethiopia; 3 Department of Public Health, ArbaMinch University, ArbaMinch, Ethiopia

**Keywords:** Tuberculosis, Delay in treatment seeking, Delayed diagnosis, Ethiopia

## Abstract

**Background:**

Tuberculosis (TB) is a major public health problem in Africa with Ethiopia being the most affected. Treatment delay is an important indicator of access to TB diagnosis and treatment. However, little is known about factors associated with treatment delay of pulmonary TB among pastoralists. Health facility based cross sectional study was conducted on 129 pulmonary TB patients in pastoralist community. The study was conducted in three health centers and a hospital. Time between onset of TB symptoms and first visit to a professional health care provider (patient delay), and the time between first visits to the professional health care provider to the date of diagnosis (provider's delay) were analyzed using SPSS 16.0 statistical software.

**Findings:**

A total of 129 new smear positive pulmonary TB patients participated in the study. The median total delay was 97 days. The median patient and health provider delays were 63 and 34 days, respectively. Ninety six percent of the patients were delayed for more than the twenty one days cutoff point. Patient delay was positively associated with first visit to traditional healer/private clinic/drug shop, rural residence, being illiterate, living in more than 10 kilometers from health facility; severity of illness at first presentation to health facility. Provider delay was positively associated with rural residence, being illiterate, patient with good functional status, patients in contact with more than two health providers, and place of first visit being traditional healer/private clinic/drug shop.

**Conclusions:**

This study showed that majority of smear positive patients delayed either for diagnosis or treatment, thus continue to serve as reservoirs of infection. This indicates that there is a need for intervention to decrease patient and provider delays. Effort to reduce delays in pastoralist communities should focus on improving access to services in rural communities, engaging traditional and private health providers and should target illiterate individuals.

## Findings

### Background

The STOP Tuberculosis (TB) strategy was introduced in 2006 to achieve the TB related Millennium Development Goals (MDG) by 2015. The STOP TB strategy focus on expansion and enhancement of quality DOTS program, addressing TB/HIV co-infection and MDR TB, health system strengthening, involving all providers including the private sector, empowering of TB patients and communities, and promote and enhance research [1]. Despite all these efforts, TB is still the major cause of morbidity and mortality. The 2010 WHO report revealed that there were a total of 9.4 million incident and 14 million prevalent cases of TB and 1.3 million deaths in 2009 [2].

To decrease the impact of TB, the United Nations included TB prevention and control among its eight Millennium Development Goals, with a proposal to reduce TB incidence to half the 1990’ level by 2015. To measure and achieve this, the World Health Assembly (WHA) highlighted two indicators: 70% global and in-country case detection rates and successful treatment of 85% of cases [3]. However, despite this increased effort invested in TB control, the epidemic rages on. More than 90% of global TB cases and deaths occur in the developing world; here 75% of cases are in the most economically productive age group (15-54years). An adult with TB loses on average three to four months of work time. This results in the loss of 20-30% of annual household income and, if the patient dies of TB, an average of 15 years of lost income [4].

TB is a major public health problem in the Horn of Africa with Ethiopia being the most affected where TB cases increase at the rate of 2.6% each year [5]. The World Health Organization (WHO) has classified Ethiopia 7th among the 22 high burden countries with TB infection in the world [6,7]. According to the WHO estimate, there incidence and prevalence of all forms of TB were 572 and 359 per 100,000 respectively. The case detection rate of all forms of TB and smear positive TB were 50% and 34% respectively [2]. An estimated 5000 MDR TB exists in Ethiopia and only 88 of them had received second-line anti-TB drugs [2,8]. However, a recent community based national survey indicated a lower prevalence of TB [9].

In Ethiopia the case detection rate for smear positive TB is only 34%. This indicates that the proportion of notified cases diagnosed as smear-positive is low in Ethiopia, and has stayed within the range 27–36% during the period 1995–2004, detecting them early and putting them on treatment and ensuring cure should be the highest priority [10,11]. Strategies aiming to reduce the time between the onset of symptoms and the initiation of effective chemotherapy may impact the infectious duration in the community and thereby reduce the number of new infections. However, factors contributing to treatment delay are potentially numerous, and likely to vary considerably in relative importance between populations in their local settings [8].

In Ethiopia many studies have documented delayed diagnosis and treatment of TB in agrarian communities; patient delay in Amhara region of Ethiopia (median, 30 days) [12], Awasa, Ethiopia (median, 31 days) [13], and Tigray region (median, 30 days) [6], in the study conducted among pastoralists of Somali region of Ethiopia the median delay was found to be 60 days [14]. Whereas the provider delays in Afar [15], Ethiopia (median, 6 days), Somali, Ethiopia (median, 6 days) [14] and Amhara region (median, 21 days) [12].

In the absence of strong information that state factors contributing to treatment delay of TB among pastoralists the importance of this study is immense. Due to the difference in lifestyle of pastoralist communities from the agrarian communities the extent and factors behind delays could be different. Most of previous studies conducted in Ethiopia focused on agrarian communities; little is known about the prevalence and associated factors of TB diagnosis and treatment delays among pastoralist communities. We performed such study to gain information on factors influencing delay to TB treatment among pastoralist in Bale zone in particular and in Ethiopia in general.

## Methods

**Figure 1:**
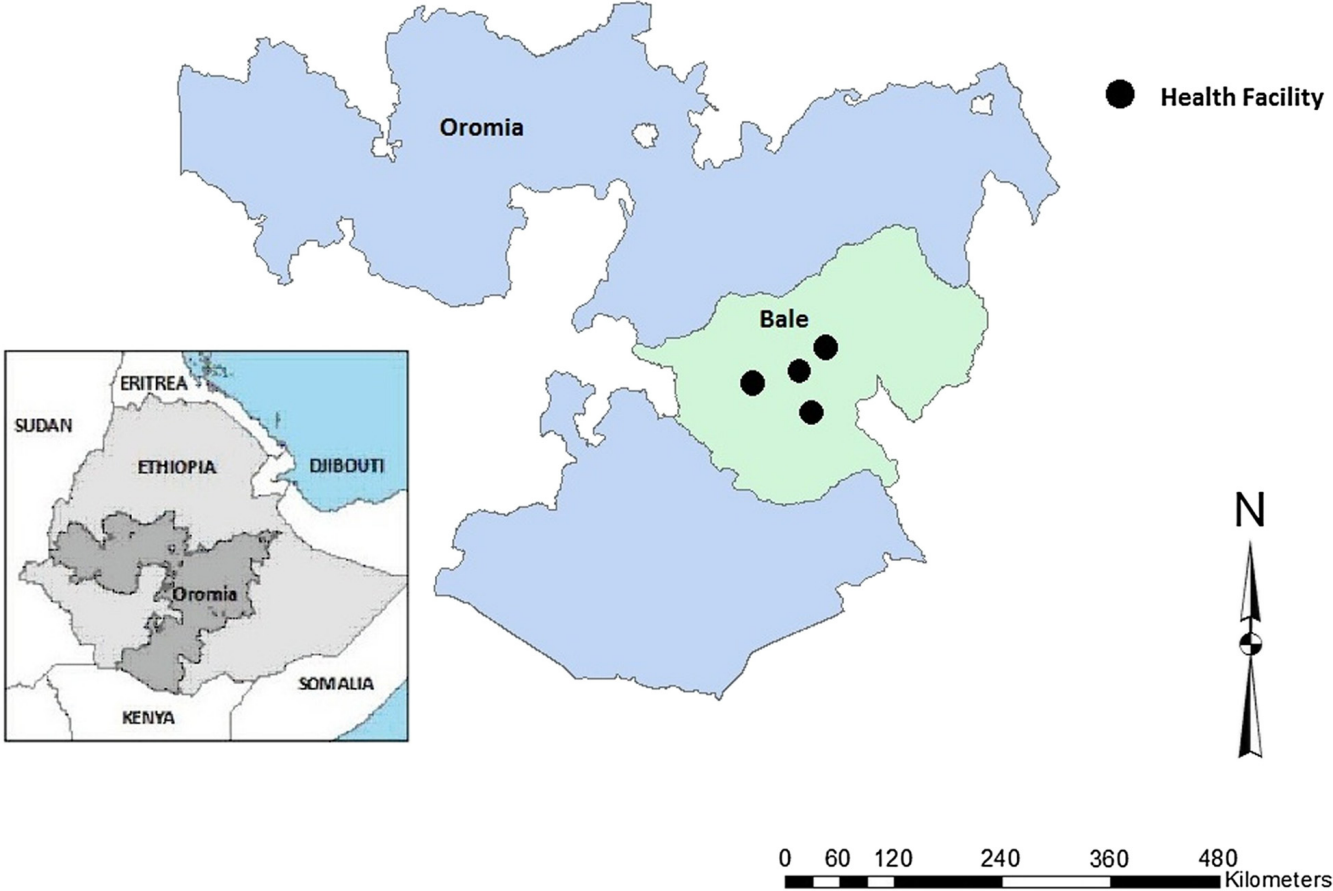
Map of the study sites, Ethiopia.

A cross-sectional survey was conducted from February to March 2011 in one hospital (Ginner Hospitals) and three health centers (Reyitu, Sewena and Dallo manna Health Centers) of the pastoralist community in Bale Zone. The zone is found in Oromia Region with total population of 1,593,298 projected in 2011 of these; 601,556 populations are pastoralist and are subdivided administratively into 19 districts and two city administration with a total of 401 Kebeles (lowest government administrative structure). Of 19 districts 9 districts are pastoralist where a hospital and 6 health centers serve as diagnostic and treatment centers for DOTS [16]. The study population**s** are all pulmonary positive TB patients on treatment for less than 15 days and newly diagnosed pulmonary positive TB patients in the selected hospital and health centers (Figure 1).

### Sampling and sample size

All pulmonary TB patients newly diagnosed during the study or on treatment for less than 15 days were included in the study. According to the zonal health department report of the previous year and three consecutive months before data collection period, it was expected that 135 pulmonary positive TB patients would be available during the study period [16].

### Selection of the health facilities

There were a hospital and nine health centers in the area. Of the total health facilities six health center and a hospital provide both diagnosis and anti-TB treatment. Of this three health centers were selected randomly and a hospital selected purposively.

For each selected health facility study subjects were allocated proportional to their patient flow based on their previous year TB patient report and three consecutive months before study period. As a result, Ginner hospital 50 patients, DalloMana health center 32 patients, Rayitu health center 27 patients and Sewena health center 26 patients.

### Data collection and tools

This study used an interviewer administered questionnaire which was adopted from similar studies done in Ethiopia and elsewhere [12,14,17,18]. Before interviewing the patients, eligible study subjects were identified by reviewing the TB registration. Information such as name of the DOTS centre, name of the diagnostic centre for sputum examination, patient's registration number (District TB number), date sputum examined and treatment started was abstracted from TB unit registers and TB treatment cards.

There were 6 nurse enumerators and three supervisors who had BSc degree in Nursing. Individuals who can speak the local languages Afan Oromo and Amharic were recruited from respective health facilities from different department. The collected information from the TB registry and the patients were reported to the supervisor every 2–3 days, to enable taking immediate action in case inconsistencies and problems happen on the reported data. Reports were crosschecked with the main TB registry found in the respective health facilities. Effort was made to contact eligible patients who were on treatment for less than 15 days of TB treatment but not visiting the health facilities during the study period. Respondents were first asked to estimate the time in days they had been experiencing cough or other symptom before presenting to health facilities with the current diagnosis of TB. A calendar based on local religious, political and agricultural events was used to collect information on date of onset of first symptoms suggestive of TB, for patient unable to estimate the date.

The questionnaire has one separate page designed to extract diagnosis and treatment information from TB registers (TB treatment cards and unit TB registers). The questionnaire contained the following questions: Demographic and socio-economic variables: age, sex, occupation, education level, and family size; disease factors: symptoms and their duration, date of first presentation to a modern health facility, results of sputum microscopy for AFB, date diagnosis confirmed, date treatment commenced, type of the patient (new case, relapse, failure, transfer in, return after default) and others ; psychological factors: patient beliefs, perceptions, stigma, attitudes and knowledge about TB; Institutional or health service factors: distance to health facilities, costs of travel, medical expenditure on treatment of TB related symptoms , reasons for delay. Each interview schedule was last approximately 40 minutes per study subjects and allow for careful probing of responses to minimize recall bias. Other data sources were case notes and referral letters used.

### Data quality control

The following measures were taken to maintain and increase the reliability and validity of the study. The questionnaire was pre tested on 10%( 14) TB patients who were not illegible for the study. Based on the pre test modifications were incorporated to the questionnaire. Since the data requirement was more importantly focusing in knowing the date for different events by the study subjects, key events after start of symptom and the date was asked in different ways to cross check with total duration the patients reported.

The enumerators and supervisors were trained for two days about administering the instruments.

Frequent follow up was done during the phase of data collection. Data was checked and entered at the field level by the investigator and cleaned before analyses. All of the entered data was checked again before final analysis.

### Data Analysis

Data were checked for its completeness every day, edited, coded, entered, cleaned and analyzed using SPSS version-16. The collected data were processed by using both descriptive and analytical method which includes tables, summery measures of central tendency (like mean and median) and dispersion (standard deviation, range), odds ratio and confidence intervals were used. Logistic regression was done for significant variables to identify factors associated with treatment delay. P-value < 0.05 was considered statistically significant. Two logistic regression models were produced to indentify factors associated with patient and provider delays.

### Analyses of knowledge about and stigma associated with TB

Subjects were asked specific questions/statements to assess their knowledge about signs and symptoms and perceived causes of TB, and stigma associated with TB. Then a separate summary indicator was developed for analyses by dividing the total score into three strata, ≤25th percentile, between 25th and 75th percentile and ≥75th percentile. Subjects who scored below or equal to the 25th percentile for knowledge questions were considered to have “unsatisfactory knowledge”, those who scored between the 25 and 75 percentile was regarded as having “average (fair) knowledge”, while those who scored above or equal to the 75 percentile was considered to be “knowledgeable” on TB. Similar procedure was used for stigma level associated with TB. Accordingly, subjects were categorized as “no perceived stigma,” “some perceived stigma” and “high perceived stigmata” [18].

### Operational definition

In this study the following operational definitions were used: Patient delay is the time interval from the appearance of the first symptom of TB until the first visit to health care facility. Thus, patients who consulted health care facilities more than 2 weeks after the onset of the constitutional signs and symptoms of TB are considered as ‘delayed’. Health provider delay: Health provider delay in this context is defined as the time ranging from patient’s first contact to any modern health facility to date of commencement of anti-TB treatment. This comprises time spent during referrals between facilities, diagnosis and time between diagnosis and start of treatment. Thus, period between reporting to a health facility and initiation of treatment for more than 7 days would be considered as ‘delayed’. Total delay is the sum of patient delay and health provider delay.

The diagnosis of TB was according to the national TB diagnosis algorism. A case of TB is a patient in whom TB has been confirmed by sputum examination or by a clinician. A pulmonary case is a patient with TB disease involving the lung. A case of smear positive pulmonary TB is a patient with at least two initial positive sputum smears, or one sputum smear positive plus radiographic abnormalities consistent with active pulmonary TB as determined by a clinician. A sputum smear negative pulmonary TB case is a patient diagnosed with pulmonary TB by a clinician not meeting the above criteria for smear-positive disease. In the context of the study TB diagnosis was based on sputum examination, radiographic abnormalities, there were no services of culture in the area.

Pastoralists are migratory people whose livelihood largely depends on livestock raising. These people move seasonally in search of pasture and water.

### Ethical consideration

The study protocol was approved by the ethical review committees of Jimma University, Official letter of co-operation was also written to Bale Zone Administration. Letter of co- operation was obtained from Zonal Health Desk to the four selected health institutions. Informed written consent was obtained from each study participants. Privacy and strict confidentiality was assured for the information provided and their names were not included in the questionnaire. Referral to counseling and testing for HIV/AIDS was done for all patients.

## Results

### Socio-demographic characteristics of the participants

A total of 136 smear positive pulmonary TB patients were registered. Seven patients were excluded from the study (5 cases were aged below 15 years and 2 case were critically ill and unable to respond). A total of 129 pulmonary TB patients were eligible for this study with ninety six percent (96%) response rate. Most of the participants (56%) were males, 66.7% found in the age group 15–35 years, 75.2% reside in rural areas, 69.0% were married, 73.6% were illiterate, Most(75.2%) were Oromo by ethnicity and 90.7% Muslim by religion and almost half (49.6%) were pastoralist, by occupation. Mean age for study participants was 35.3 with standard deviation of 14.32. The average monthly family income was 300.58 Eth. Birr (1USD = 13ETB). Among the study subjects 89(69%) lived beyond 10 km from a health facility. (Table 1)

**Table 1:** General characteristics of pulmonary Tuberculosis patients (n = 129) in pastoralist community in Bale Zone, April 2011

**Patient characteristics**		**Frequency**	**Percent**
**Age (years)**	15 – 25	48	37. 2
	26 – 35	38	29.5
	36 – 45	21	16.3
	> 45	22	17.1
**Sex**	Male	73	56.6
	Female	56	43.4
**Place of residence**	Rural	97	75.2
	Urban	32	24.8
**Marital Status**	Single	31	24.0
	Married	89	69.0
	Divorced/separated	4	3.1
	Widowed	5	3.9
**Religion**	Orthodox Christian	11	8.5
	Muslim	117	90.7
	Catholic	1	0.8
**Ethnicity**	Oromo	97	75.2
	Amara	8	6.2
	Somale	24	18.6
**Education**	Illiterate	95	73.6
	Read and write	8	6.2
	Primary (1–4)	16	12.4
	Junior (5–8)	3	2.3
	Secondary (9–12)	6	4.7
	12+	1	0.8
**Family size**	1 to 3 person	11	8.50
	4 to 6 person	55	42.60
	> 6 persons	63	48.80
**Distance from health facility**	≤10 Km	37	28.6
	>10 Km	92	71.4

### Clinical characteristics and health seeking patterns of the patients

The major signs and symptoms that patients experienced during the onset of their illness were cough for more than 2 weeks (94.5%), followed by night sweating (89%), loss of appetite (85.3%), and fever for more than 2 weeks (72.1%), weight loss (69.7%), chest pain (58.1%) and bloody sputum (30.2%).

Concerning first place of contact for seeking help 32% of patients reported first to Drug shop/pharmacy or private health facility. Significant (30%) patients reported to traditional healers before they visited any other conventional health facilities. The first place of contact for seeking help was different between urban and rural patients (p-value = 0.026) educated and non-educated (p-value < 0.001). There was no significance difference in first place of contact with respect to gender and income. Concerning reasons that made the patient choose the first conventional health provider were, 56.6% patients chose because the facility/provider was found nearer, 28.7% because of better and quality services they assumed to get, 8.5% because of fair price charged for services, 6.2% mentioned that because of good contact and hospitality attracted them.

Up on first presentation to a health care provider, 34.9% of symptomatic patients were given non-anti-TB drugs, while sputum for acid fast bacilli test was taken for only 24.8% of the symptomatic patients, 20.9% referred to other health facility and only 19.4% started anti-TB drug.

### Respondents' general knowledge of TB and stigma associated with TB

More than half of the study participants (65%) heard about TB before. Small proportion of respondents (8.5%) knew microbes cause TB disease. The majority (61.2%) of the respondents had unsatisfactory (poor) knowledge on TB, while 33.3% had average or fair knowledge and the rest 5.4% were knowledgeable about TB.

Regarding the level of stigma associated with TB, 3.1% told for nobody about their illness, most of them informed family members and close confidants. The majority (70.0%) of the respondents experienced no stigma, while 24.0% some stigma and the rest (6.0%) were highly stigmatized because of TB.

### Patient delay

The median delay from onset of symptoms to first visit of a health provider was 63 days, range (14–896 days). Patient delay has contributed 53% to the overall total delay to treatment. However, only 11.6% of patients were able to report within the first two weeks (acceptable delay) after developing symptoms of pulmonary TB whereas, for 89.9% of the respondents it took more than 14 days. The longest delay was reported to be 2.5 years.

About 90% of patients, who delayed seeking health care for more than 14 days, were asked for reasons of their delay, 20.9% of patients delayed assuming that symptom will disappear by itself, while 22.5% delayed because of shortage of money for transportation and health care, 12.4% following traditional/spiritual treatment and 7.8% reasoned that health facilities were too far. Some of them mentioned other reasons, like workload (7.0%), money was not provided on time from family (4.7%), lack of transportation (7.0%), afraid of long processes in health facilities (2.3%), and the rest (15.4%) provided no specific reason.

In the logistic regression patients who lived beyond 10 kilometers radius of a health facility were 2.5 times more likely to delay compared to those lived within 10 kilometers radius (AOR = 2.50, 95%CI 1.67–9.44). Illiterate patients were 2.6 times more likely delay compared to their counterparts (AOR = 2.65, 95%CI 1.72-9.73). Patients working some times before illness were delay compared to those severely ill (bedridden) (AOR = 15.1, 95%CI 2.84-80.37). Place of first visit being traditional healer, drug shop, private clinic and health post, were 12 times more likes delay compared to those first visit health center and hospitals(place of diagnosis) (OR = 12. 2, 95%CI 2.80-53.11) (Table 2).

**Table 2:** Factors associated with patient delay among pulmonary Tuberculosis patients in pastoralist community, Bale zone, April 2011

**Patient characteristics**		**Patient delay**		**COR(95%CI)**	**AOR¥ (95%CI)**
		Yes*(%)	**No (%)**		
Sex	Female	52(92.9)	4(7.1)	2.3(0.69-7.67)	0.44(.11-1.78)
	Male	62(84.9)	11(15.1)	1.00	1.00
Residence	Rural	90(92.8)	7(7.2)	**4.3(1.40-13.00)**	**5.1(1.39-18.90)**
	Urban	24(75)	8(25)	1.00	1.00
Place of first visit	Traditional healer, Drug shops, private clinic and health post	92(96.8)	3(3.2)	**16.7(4.34-64.39)**	**12.2(2.80-53.11)**
	Health Center and Hospital	22(64.7)	12(35.3)	1.00	1.00
Marital status	Married	89(90.8)	9(9.2)	2.4(0.77-7.3)	1.4(0.31-5.87)
	Un married	25(80.6)	6(19.4)	1.00	1. 00
Educational status	Illiterate	88(92.6)	7(7.4)	**3.9(1.28-11.60)**	**2.7(1.22-9.73)**
	Literate	26(81.3)	8(18.7)	1.00	1.00
Family income (Birr)	≤ 100 per month	11(84.6)	2(15.4)	1.2(.098-`14.69)	0.3(.02-9.42)
	100 to 300 per month	43(82.6)	9(17.4)	.48(.094-2.41)	0.03(0.04-2.63)
	300to 500 per month	39(92.8)	3(7.2)	1.3(0.20-8.42)	0.4(0.04-4.93)
	>500 per month	20(90.9)	2(9.1)	1.00	1.00
Distance from the HF to home	>10 Km	85(92.4)	7(7.6)	**3.4(1.12-10.05)**	**2.5(1.01-9.44)**
	≤10 Km	29(73.4)	8(26.6)	1.00	1.00
Smoking	Non-smoker	65(83.3)	13(16.7)	1.00	1.00
	Current smoker	49(96.1)	2(3.9)	**4.9(1.06-22.74)**	3.5(0.64-18.50)
Level of severity before first visit to health facility	Working full time	20(95.2)	1(4.8)	3.4(0.67-66.82)	2.2(0.37-12.90)
	Working some times	65(98.5)	1(1.5)	**11.4(2.37-54.39)**	**15.1(2.84-80.37)**
	Bedridden	31(73.8)	11(26.2)	1.00	1.00
Sold their assets to go health facility beforefirst visit	Yes	67(91.8)	6(8.2)	**8.4(1.78-19.5)**	**6.67(3.81-11.99)**
	No	49(89.1)	6(10.9)	1.00	1.00

Patient delay: ≥ 2 weeks, COR = Crude Odds Ration, AOR = Adjusted Odds Ratio, ¥ adjusted for variables in the table.

### Health provider delay

The median health provider delay was 34 days (range 8–105) days. The main reason for the diagnosis delay were, advice or other drug was given rather than anti-TB treatment, lack of reagent and delay of sputum examination in laboratory, and long appointment time to return to health facility. Concerning the place of TB diagnosis 55.8% was confirmed at hospital level, 44.2% were in health centers that were closer to the community. After confirmation of TB diagnosis, median treatment delay of 2 days was observed (mean = 2.1 days), the maximum observed treatment delay was 11 days. Forty seven percent of total delay was contributed by health provider delay. The last diagnosing facility delay contributed 17.8% to the total health system delay, while days spent after TB diagnosis (treatment delay), contributed 6.3% to health system delay.

In the logistic regression patients who lived in rural area were 2.9 times more likely delayed compared to those who live in urban areas (AOR = 2.87, 95% CI 1.01-8.10). Patients who were illiterate were 3.4 times more likely delay compared to those literate patients (AOR = 3.47 95%CI 1.16-10.30). Patients with good functional status were 2.2 times delayed compared to those severely ill (bedridden) (AOR = 2.18, 95% CI 1.13-4.32). Patients who visit more than 2 different types of health service providers before the start of anti-TB treatment were 3.2 times more likely delayed compared to those with 2 or less (AOR = 3.2, 95% CI 1.2-7.86). Patients who first visited drug shop, private clinic and health post, were more likes delayed compared to those who first visited health center and hospitals (place of diagnosis) (OR = 19. 7; 95%CI 12.63-34.52) (Table 3).

**Table 3:** Factors associated with provider delay among pulmonary Tuberculosis patients in pastoralist community, Bale zone, April 2011

**Patient characteristics**		**Provider delay**		**COR (95%CI)**	**AOR ¥(95%CI)**
		Yes** (%)	**NO (%)**		
**Gender**	Female	47(83.9)	9(6.1)	1.59(.65-3.88)	1.28(.40-4.06)
	Male	56(76.7)	17(23.3)	1.00	1. 00
**Residence**	Rural	83(85.6)	14(14.4)	**3.74(1.49-9.38)**	**2.87(1.01-8.10)**
	Urban	19(61.3)	12(38.7)	1.00	1.00
**Marital status**	Married	77(78.6)	21(21.4)	0.71(0.24-2.06)	0.39(.09-1.74)
	Un married	26(83.9)	5(16.1)	1.00	1.00
**Educational level**	Illiterate	80(84.2)	15(15.8)	**2.55(1.03-6.31)**	**3.47(1.16-10.30)**
	Literate	23(67.6)	11(32.4)	1.00	1.00
**Family income** (Birr)	≤ 100 per month	11(84.6)	2(15.4)	0.87(.13-6.03)	.54(.04-7.47)
	100 to 300 per month	42(80.7)	10(19.3)	. 66(.166-2.67)	.46(.08-2.56)
	300 to 500 per month	31(73.8)	11(26.2)	.45(.11-1.80)	.47(.09-2.50)
	>500 per month	19(86.4)	3(13.6)	1.00	1.00
**Distance from the facility**	>10KM	77(83.7)	15(6.3)	2.17(.89-5.32)	1.41(0.42-4.71)
	≤10KM	26(72.3)	11(27.7)	1.00	1.00
**Level of severity before illness**	Working full time	17(80.9)	4(9.1)	3.9**(1.21-5.95)**	**2.18(1.13-4.32)**
	Working some times	52(78.8)	14(21.2)	3.4(.22-7.76)	2.48 (.15-4.32)
	Bedridden	22(52.4)	20(47.6)	1.00	1.00
**Tuberculosis patient contact**	Yes	29(93.5)	2(6.5)	**4.7(1.04-21.18)**	2.73(0.21-14.62)
	No	74(75.5)	24(24.5)	1.00	1.00
*Types of different health service provider contact till start of AntiTB treatment	≤ 2 types	22(53.7)	19(46.3)	1.00	1.00
	> 2 types	76(86.4)	12(13.6)	**5.4(1.54-9.21)**	**3.2(1.2-7.86)**
**Types of health facility first visited by patients**	Health Center/Government hospital	35(48.6)	37(51.4)	1.00	1.00
	Health post /private clinic /drug shop	53(94.6)	3(5.4)	**18.7(12.46-28.84)**	**19. 7(12.63-34.52)**

* Doesn’t include repeated visits patients made to one health provider, ** Provider delay: ≥ 1 week. COR = Crude Odds Ration, AOR = Adjusted Odds Ratio, ¥ adjusted for variables in the table.

### Total delay

The median total delay was 97 days. Only 3.1% of the total respondents were detected and put on treatment within twenty one day of the onset of their illness whereas for 96.1% of the respondents treatment was started after twenty one days. The major proportion (53%) of the total delay was attributed to patient delay.

The choice of first health care provider appeared to influence the median total delay to treatment Patients who first visited government hospital reported the shortest delay from first visit to treatment (median 35 days, n = 26), while those who first visited traditional or spiritual healer reported the longest delay (median 90 days, n = 38). It was also, observed that people of higher education started treatment early compared to patients of lower educational status (p = 0.006).

## Discussion

Early diagnosis of disease and prompt initiation of treatment is essential for an effective TB control program. Delay in diagnosis may worsen the disease, increase risk of poor clinical outcome, including death and enhance transmission of TB in the community [19,20]. In this study, the median total delay was 97 days. The median patient and health provider delay were 63 and 34 days, respectively. Ninety six percent of the patients were delayed for more than three weeks recommended to detect suspected TB. The delays are attributed to alternative treatment, health workers' low index of suspicion, access to diagnosis and treatment and patient characteristics.

The patient delay from onset of symptoms to first visit of a health provider ranges from 14 to 896 days (median of 63 days) which is much longer than the acceptable duration of two weeks recommended by the WHO for suspected TB cases [7]. The delay in our study is comparable with Somali region Ethiopia [14] (median, 60 days) and Addis Ababa (median, 60 days) [21]. However it is much higher than, from Amhara region of Ethiopia (median, 30 days) [12], Awasa, Ethiopia (median, 31 days) [13], and Tigray region (median, 30 days) [6]. In addition the delay is much higher than that reported from other African countries Cameron (median, 14 days) [22], Zambia [23] and Gambia [24].

The difference observed with most studies could be due to the difference in the population characteristics. Our study focused on pastoralist communities similar to the study conducted in Somali region of Ethiopia [14]. The population is characterized by mobility and illiteracy; in this study 73.6% of patents are illiterate and have poor accesses to health facilities. Access to health care is defined in Ethiopia as living within 10 km radius to health facility. In this study 71.4% of the patient live out of 10 kilometers radius from health facility, hence lacked access to health care. Previous study has documented that pastoralist communities are more marginalized than the settled rural residents [14].

Patient delay significantly varied with the patients’ area of residence. Patients from rural had high median delay compared to those from urban. In addition patients with close proximity to health facility had lower delays. Patients that initially visited non-formal health care providers had longer median patients' delay compared to those who went directly to health provider. This was similar to study done in northern part of Ethiopia [12]. All these factors add up to the importance of access to health information and health facilities. In particular to this study DOTS services which take into account the unique life style of pastoralist community are important. In addition understanding the migratory pattern of the pastoralist community would give a better idea of where to locate health facilities for such services. One critical observation here could be considerable number of patients seek treatment from non-formal health sector and private health providers. Inclusion of the non-formal health providers as referral allies and inclusion of private sector in the TB-DOTS program can significantly reduce both diagnosis and treatment delays. In Ethiopia there are initiatives to foster public private partnership for TB treatment in major towns; therefore expansion of these services into the remote rural areas is a timely agenda.

The major reasons mentioned by patients for delay in treatment seeking were knowledge related, access and financial problems. Similar findings were reported from Nigeria [25]. This highlights the importance of decentralized TB treatments services which address the challenges faced by the pastoralist community.

On the other hand, sex and level of knowledge about disease and stigma attached to TB patients did show no significant difference on time of presentation to health care providers it differ from a study done in Nigeria [25], other several studies showed stigma attached to the TB patient as contextual factor associated to delayed seeking of the health services [20,26] which is not the case in our study.

The median provider delay from first presentation to the health facility until start of Tb treatment was 34 days, (range 7–105). This was lower than report from Ghana (median, 56 days) [27] but much higher than the findings of studies in Addis Ababa [21], Ethiopia (median, 6 days), Somali, Ethiopia (median, 6 days) [14] and Amhara region (median, 21 days) [12]. This difference might be due to low index of suspicion to TB symptoms by health providers the area is characterized by less experienced health providers. Experienced health providers tend to migrate to urban settings and to areas better-off in infrastructure. In addition supply shortage for diagnosis of smear positive TB contributes to the delays.

Most (55.8%) of TB diagnoses were confirmed at hospital level than health centers or clinics that were closer to the community, while only 9.3% of the patients visited hospital at their first visits. Although, all of the health facilities included in this study were entitled to provide the diagnostic and treatment services, the participation of health centers was very limited in contributing to the case detection. This is unacceptable when we look that most of the patients (56.1%) were following the current treatment in health centers after they were referred back from hospitals. Similar situation was reported by another study from Ethiopia [12] where most diagnosis of TB was made in district hospitals.

The current study confirms that those who visited more than two health facility had high provider delay. This is in consistent with study conducted in Zambia, where multiple health seeking encounters contributed to the prolonged duration of health service delay along with the associated medical costs [28]. In addition patients who visited health facilities with no diagnostic facilities were more delayed. In our study majority of patients with suggestive symptoms of TB were not examined correctly for TB or referred to diagnostic facilities up on their first arrival, rather most (34.1%) of them were given treatment for diseases other than TB. This highlights the importance of establishing functional referral pathways between first encounter points and point of diagnosis. In our context building the capacity of health providers at first level health facilities and ensuring supply chain management could contribute to the reduction of delays in diagnosis and treatments.

Patients’ characteristics such as education and rural residence were associated with the provider delays. All this factors contribute to the biomedical knowledge of patients on TB. Severity of the illness was also another factor contributing to delays. This might indicate that the pastoralist community seeks treatment for at an advanced disease stage.

The current study has limitations: recall bias might be there as patients were asked about events which happened sometimes back. However, we have included patients which were on treatment for not more than two weeks to reduce the recall bias.

## Conclusions

The study identified extreme patient and provider delays among pastoralist communities in Southeast Ethiopia. The factors associated with patient delay were rural residence, being illiterate, low access to health care facilities, and first visit to non-formal or first level facilities and private providers.

Factors associated with provider delay include rural residence, being illiterate, health post /private clinic /drug shop being first contact point and having visited more than two health care providers. To increase case detection establishing functional referral pathways, building the capacity of health providers to increase the index of suspicion and availing rapid diagnostic test for TB at lowest health facilities are important first step. Inclusion of non-formal health sectors and private providers in the process of case detection and identification could contribute in reducing delays. To increase access to DOTS program by pastoralist community it is imperative to establish treatment centers which takes into consideration the lifestyle of the community and follows migratory patterns. Finally improving awareness of the community is very important to induce early treatment seeking behavior among pastoralist communities.

## Competing interest

The authors declared that there is no competing interest.

## Authors’ contributions

AH participated in design of the study, drafted the manuscript, coordinated the field work and analyzed the data. AD participated in the design of the study, helped to draft the manuscript and review of article. FT participated in design of the study, analysis and review of article. SM participated in field work, analysis and review of article. KD and SB drafted and reviewed the manuscript. All authors read and approved the final manuscript.
